# Supplementary material for: All-optical ultrafast ReLU function for energy-efficient nanophotonic deep learning
Source: Nanophotonics. 2022 May 2;12(5):847–55. doi: 10.1515/nanoph-2022-0137 (PMC11501931; doi:10.1515/nanoph-2022-0137)
Supplement: Supplementary file 1 — Supplementary Material Details [file j_nanoph-2022-0137_suppl.pdf]

All-optical ultrafast ReLU function for energy-efficient nanophotonic deep learning: supplemental document

This document provides supplementary information to "All-optical ultrafast ReLU function for energy-efficient nanophotonic deep learning."

1. EXPERIMENTAL SETUP

The experimental setup for the optical ReLU measurements is depicted in Fig. S1. The pump laser is a mode-locked Yb-fiber laser which provides 70 fs pulses at 1045 nm with up to 1 W average power at a 250 MHz repetition rate (Menlo Systems Orange A). The laser output is then split into two paths. The first path is sent to a synchronously pumped degenerate optical parametric (SPDOPO) oscillator based on periodically-poled lithium niobate (PPLN) which is used to efficiently generate pulses at 2090 nm[1]. The OPO is locked using a "dither and lock" scheme, facilitated by the Lock-In + PID application for Red Pitaya [2, 3]. A variable ND filter is added to the output of the OPO to control the 2090 nm power sent to the device. The second 1045 nm path is sent to a delay stage. Course adjustment of the delay is done through manual tuning of the stage position and micrometer arm while fine adjustment is performed using a piezoelectric actuator. This delay enables temporal overlap of the two paths, and fine adjustment is used to change the relative phase of the fundamental and second harmonic for the OPA process. Like the other path, a variable ND filter is also placed along this path for adjusting the 1045 nm power. The two paths are recombined at a dichroic mirror with high transmission at 1045 nm and high reflectivity at 2090 nm before going to the device.

Focusing to and coupling from the device is done using a reflective objective (Newport 50102-02). Temperature tuning of the device for fine adjustment of the quasi-phase matching condition is done using a thermoelectric cooling stage (TEC). The output of the chip is short-pass filtered around 1700 nm to remove all remaining signal at 2090 nm and then split into two paths. The signal on one path is measured with a detector and used for feedback to the delay stage. A "dither and lock" scheme, similar to that used for the OPO, is employed here to lock the relative phases of the two inputs to switch between amplification and deamplification in the OPA process [2, 3]. The second path is coupled to fiber and sent to an optical spectrum analyzer (OSA) for measuring the output power and spectrum (Yokogawa AQ6370D).

2. DEVICE FABRICATION AND CHARACTERIZATION

For our devices, a wafer with 700 nm of X-cut MgO-doped LN on top of 2 μm of SiO_2 was used. 15 nm of Cr underneath 55 nm of Au were then e-beam evaporated and patterned via e-beam lithography to form poling electrodes. 300V pulses were used to pole the chip, and the quality was confirmed using second harmonic microscopy. Waveguides were subsequently patterned on the chip using hydrogen silsesquioxane (HSQ) as the e-beam resist and 15 nm of Ti as an adhesion layer. They were dry etched with Ar^+ plasma in an inductively-coupled plasma reactive-ion etcher (ICP-RIE), and the remaining resists and side-wall re-deposition were removed using Buffered oxide etchant (BOE) and RCA-1. Finally, the waveguide facets were mechanically polished.

In Fig. S2, we display the measured spectrums of 2ω, ω and their non-linear interaction in the waveguide. (a) and (b) show the input spectrums of 2ω and ω, respectively and (c) shows the evolution of 2ω pulse as the phase difference is modulated. We can see that for the positive signal values corresponding to the phase relationship $2\phi_\omega - \phi_{2\omega} = \pi/2$, the 2ω signal grows due to SHG process while depleting the ω pulse. On the other hand, for the phase relationship $2\phi_\omega - \phi_{2\omega} = -\pi/2$, the ω pulse grows due to optical parametric amplification, thereby depleting the 2ω as evident from the dip in the spectrum.

We now estimate the input and output coupling efficiencies of our device. A detailed discussion is provided in previous work on optical parametric generation (OPG) and amplification (OPA) [4, 5]; here we outline the main steps. For a degenerate OPG process in the high parametric gain

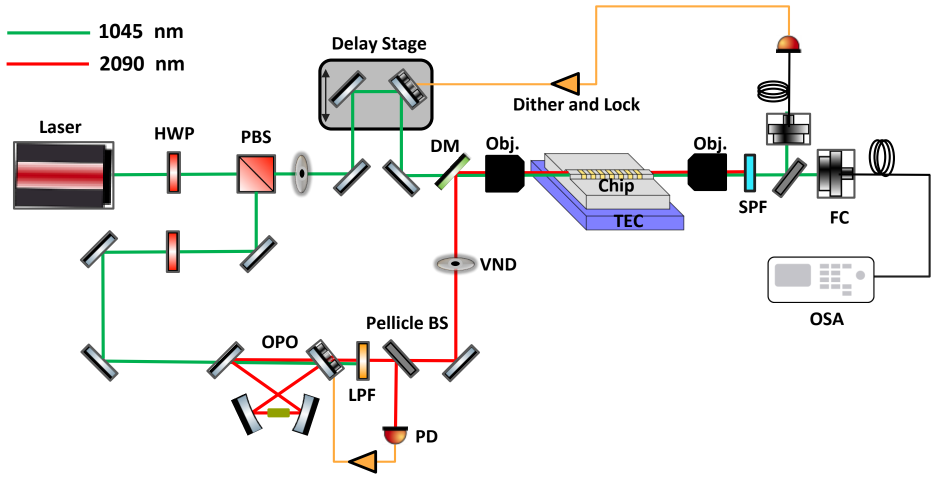

Fig. S1. Experimental Schematic for all-optical ReLU measurements. The pump laser at 1045 nm is first split into two paths. One beam is used to pump our SPDOPO above threshold generating signal at centered at 2090 nm. The other beam is guided to a delay stage and further overlaps with the 2090 nm OPO signal at a dichroic mirror. Both beams are then coupled in and out from the chip using high NA reflective objectives. Next, the waveguide output is filtered with a short pass filter for filtering out the 2090 nm followed by splitting 1045 nm into two paths. Both of the 1045 nm beams are coupled into multimode fibers; one beam is measured by the OSA while the other beam is used to lock the delay stage. PBS: Polarizing beamsplitter, HWP: Half-wave plate, DM: Dichroic mirror, Obj.: Reflective objective, VND: Variable neutral-density filter, LPF: Long-pass filter, SPF: Short-pass filter, FC: Fiber Coupler, OSA: Optical spectrum analyzer, PD: Photodetector, OPO: Optical parametric oscillator.

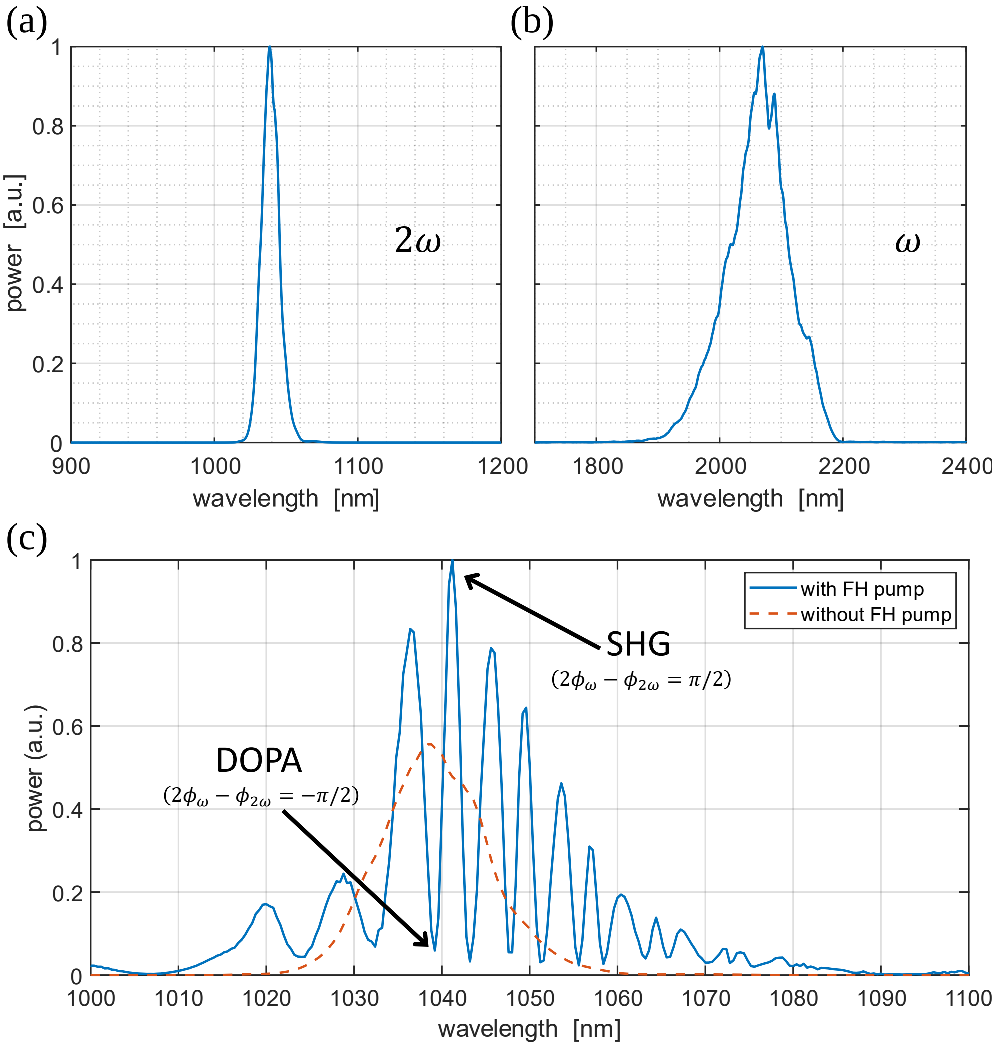

Fig. S2. Measured spectrums of ω and 2ω. In (a) and (b) correspond to the waveguide input 2ω and ω, respectively. (c) shows the evolution of the waveguide output 2ω as the phase difference between 2ω and ω is modulated.

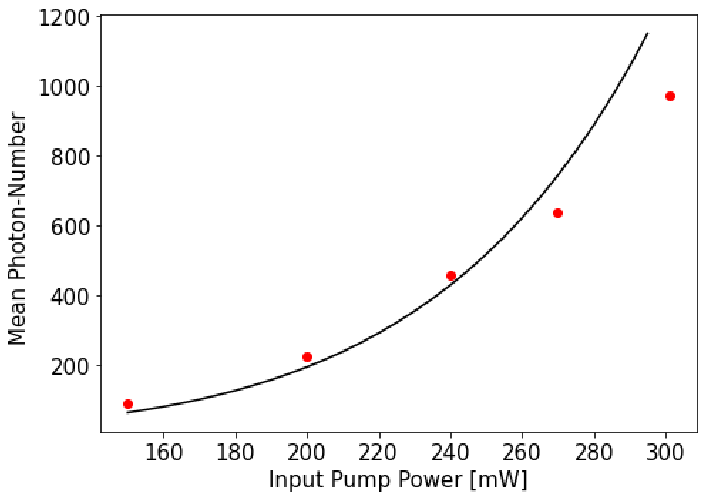

Fig. S3. Number of signal photons as the input pump power is varied. The red points are experimentally measured data for several values of pump power and the black curve shows the exponential fit used for estimating the output coupling efficiency, i.e., the η_1 parameter in Eq. S2.

regime, the generated average photon-number in an ideal case is given by

$$\langle N \rangle \approx \frac{1}{4} e^{2L\sqrt{\eta P}}, \tag{S1}$$

where P, L and η are pump power, interaction length, and non-linear interaction efficiency. In the presence of experimental imperfections such as off-chip coupling, coupling to optical fibers, and detection inefficiencies, the average photon-number is given as

$$\langle N \rangle \approx \frac{\eta_1}{4} e^{2L\sqrt{\eta_2 P}}, \tag{S2}$$

where all optical losses on the OPG signal are combined in η_1 parameter and η_2 quantifies the non-linear interaction strength and the input coupling efficiency of our second harmonic signal. From our measured data for OPG power, we determine the average photon number for various values of the second harmonic pump. By fitting the data, we can extract the η_1 and η_2 parameters. In Fig. S3, the measured average number of photons are displayed with respect to the input pump power. From the fit, we extract $\eta_1 \approx 0.20$, i.e., the estimated output coupling loss is about 7 dB, which shows a good agreement with our previous paper [4, 5]. Given the total coupling loss measured at low power, we then determine the input coupling loss as the difference between total and output coupling losses. We note that coupling losses < 1 dB per facet have been reported for thin-film lithium niobate photonics [6], which is promising for large-scale circuits.

3. SIMULATION METHOD

We numerically solved an analytical nonlinear envelope equation (NEE) in the frequency domain using a split-step Fourier technique to simulate the pulse propagation and nonlinear dynamics in the waveguide. The nonlinear step was implemented using fourth-order Runge-Kutta method. We obtained the NEE by ignoring counter-propagating modes, which are usually phase mismatched, and assuming a constant nonlinear coefficient across the entire simulation bandwidth. The fundamental and second harmonic pulses were assumed to have a transform-limited, hyperbolic-secant profile. The NEE is given by:

$$\frac{\partial A}{\partial z} = -i\left[\beta(\omega) - \beta_0 - \frac{\Omega}{v_{ref}} - i\frac{\alpha}{2}\right] A - \frac{i\omega\varepsilon_0 X_0}{8} d(z)\mathcal{F}_\Omega\left\{a^2(z,t)e^{j\phi(z,t)} + 2a(z,t)a^*(z,t)e^{-j\phi(z,t)}\right\}, \tag{S3}$$

where $A(z,\omega)$ is the complex amplitude of the field during propagation, $a(z,t)$ is the time domain representation of $A(z,\Omega)$, $\phi(z,t) = \omega_0 t - (\beta_0 - \omega_0/v_{ref})z$, β_0 is the waveguide propagation

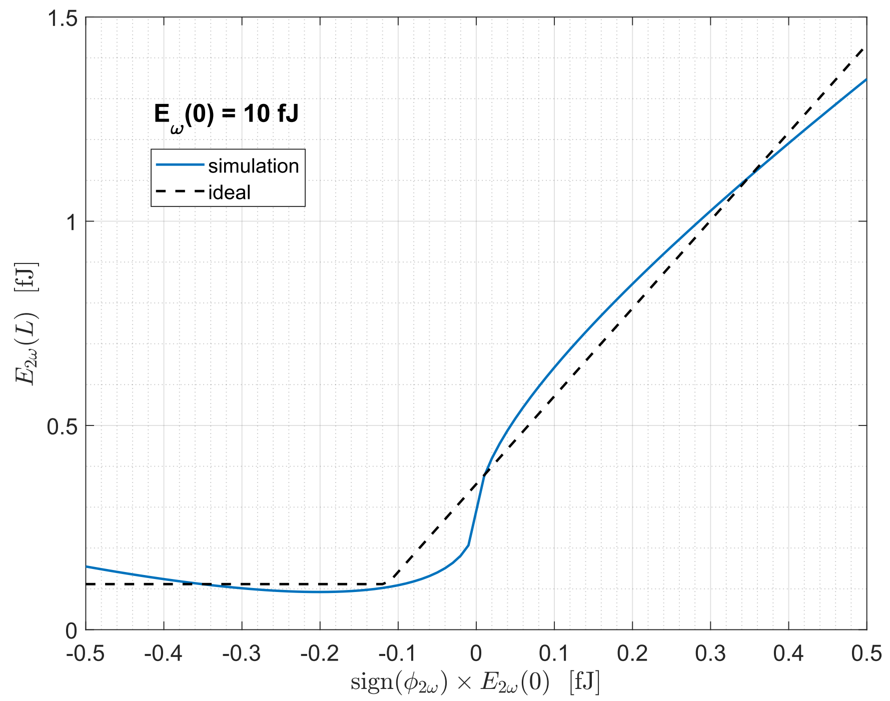

Fig. S4. Simulated ReLU-like nonlinear activation function with sub-femtojoule energies achieved using bias pulse energy of $E_\omega(0) = 10$ fJ and ideal PPLN parameters.

constant at frequency ω_0, $\Omega = \omega - \omega_0$ is the envelope frequency, ω is the optical frequency, α is the attenuation constant, $d(z) = \pm 1$ is the instantaneous sign of the nonlinear coefficient due to quasi-phase matching, $\mathcal{F}_\Omega$ is the Fourier transform in Ω, and X_0 is the effective nonlinear coefficient.

To simulate the ReLU response of our experimental device, shown in Fig. 3 in the main text, we assumed $\alpha \approx 0.1$ dB/cm and used the following waveguide geometry obtained from atomic force microscope measurements: waveguide top width of 1768 nm, ridge height (etch depth) of 377 nm, and a total lithium niobate thin-film thickness (before etching) of 713 nm. We use the effective nonlinear coefficient as a fitting parameter to match the experimental data and inferred a value of $X_0 \approx 0.36 \times 10^{-12}$ V^2, which is about $\sim 1/3$ of its ideal value.

Given the fabrication error and imperfect phase-matching of our device, we could only experimentally achieve an energy of ~ 16 fJ per activation. However, Fig. S4 shows the simulated ideal performance of a PPLN with length $L = 2.5$ mm, ridge top width of $w = 1700$ nm, etch-depth of $h = 350$ nm, and bias pulse energy of $E_\omega(0) = 10$ fJ. We see that it can achieve a ReLU-like function with sub-femtojoule energy per activation.

4. FITTING OF PUMP-PROBE SIGNAL

The input autocorrelation was fit using a Gaussian profile:

$$G(t) = \frac{1}{\sigma\sqrt{2\pi}} e^{\frac{-t^2}{2\sigma^2}}, \tag{S4}$$

where σ is related to the FWHM by $FWHM = 2\sigma\sqrt{2\ln 2}$. The exponential function with characteristic decay time of $\tau = 1/\lambda$ is defined as:

$$F(t) = e^{-\lambda t}. \tag{S5}$$

The convolution between $G(t)$ and $F(t)$ is defined as:

$$I(t') = F(t) * G(t) = \frac{1}{\sigma\sqrt{2\pi}} \int_0^\infty e^{-\lambda t} e^{-\dfrac{(t'-t)^2}{2\sigma^2}} \, dt \, . \tag{S6}$$

We fit the pump-probe signal with exponential growth and decay functions for positive and negative time delays, respectively, convolved with the input autocorrelation by using the analytical formula for Eq. S6:

$$I(t') = \frac{1}{2} e^{-\lambda(t'-\sigma^2\lambda/2)} \left[1 + \text{erf}\left(\frac{t'-\sigma^2\lambda}{\sqrt{2}\sigma}\right)\right] \, , \tag{S7}$$

where $\text{erf}(x) = \frac{2}{\sqrt{\pi}} \int_0^x e^{-z^2} \, dz$ is the error function.

5. CONVOLUTIONAL NEURAL NETWORK ARCHITECTURE

The pretrained convolutional neural network (CNN) architecture is shown in Fig. S5. The CNN was trained on the MNIST handwritten digits image classification [7] using stochastic gradient descent with momentum (SGDM) with initial learn rate of 0.01 and batch size of 128. For fine-tuning after the ideal ReLU layers were replaced with custom layers representing the experimentally measured ReLU response, the initial learn rate was decreased to 0.001.

	Name	Type	Activations	Learnables
1	imageinput<br>28×28×1 images with 'zerocenter' normalization	Image Input	28×28×1	-
2	conv_1<br>8 3×3×1 convolutions with stride [1 1] and padding 'same'	Convolution	28×28×8	Weights 3×3×1×8<br>Bias 1×1×8
3	batchnorm_1<br>Batch normalization with 8 channels	Batch Normalization	28×28×8	Offset 1×1×8<br>Scale 1×1×8
4	relu_1<br>ReLU	ReLU	28×28×8	-
5	maxpool_1<br>2×2 max pooling with stride [2 2] and padding [0 0 0 0]	Max Pooling	14×14×8	-
6	conv_2<br>16 3×3×8 convolutions with stride [1 1] and padding 'same'	Convolution	14×14×16	Weights 3×3×8×16<br>Bias 1×1×16
7	batchnorm_2<br>Batch normalization with 16 channels	Batch Normalization	14×14×16	Offset 1×1×16<br>Scale 1×1×16
8	relu_2<br>ReLU	ReLU	14×14×16	-
9	maxpool_2<br>2×2 max pooling with stride [2 2] and padding [0 0 0 0]	Max Pooling	7×7×16	-
10	conv_3<br>32 3×3×16 convolutions with stride [1 1] and padding 'same'	Convolution	7×7×32	Weights 3×3×16×32<br>Bias 1×1×32
11	batchnorm_3<br>Batch normalization with 32 channels	Batch Normalization	7×7×32	Offset 1×1×32<br>Scale 1×1×32
12	relu_3<br>ReLU	ReLU	7×7×32	-
13	fc<br>10 fully connected layer	Fully Connected	1×1×10	Weights 10×1568<br>Bias 10×1
14	softmax<br>softmax	Softmax	1×1×10	-
15	classoutput<br>crossentropyex with '0' and 9 other classes	Classification Output	-	-

Fig. S5. Pretrained convolutional neural network architecture.

6. POTENTIAL INTEGRATED PHOTONIC NEURAL NETWORKS

A promising approach to integrating the all-optical ultrafast ReLU into a complete ONN is to monolithically integrate it with high-speed electro-optic modulators in thin-film lithium niobate nanophotonic circuits. Fig. S6 shows two examples of how this can be accomplished. One method, shown in Fig. S6(a), uses a spatially-multiplexed approach. It consists of a mesh of Mach-Zehnder inteferometers, akin to those demonstrated in silicon photonics [8], to perform linear operations, directly followed by an array of PPLNs to perform the ReLU activations. Therefore, in this approach, each neuron represents a separate PPLN and the entire neural network layer is computed in a constant time step. Subsequent layers are identical in structure and can be directly cascaded following the array of PPLNs. The bias pulse can be directly fed to each PPLN using out-of-plane couplers as shown in Fig. S6(a), or by using in-plane photonic crossbar switches. The bias and signal pulses can be decoupled using wavelength-division multiplexing (WDM) filters either on-chip or off-chip.

The second method, shown in Fig. S6(b), uses a time-multiplexed approach based on a single photonic neuron folded in time with feedback-modulated delay loops [9]. In this architecture, each delay loop at each time step represents a different synaptic connection in the neural network layer. By properly updating the feedback modulators at each time step, the required linear operations can be achieved. Therefore, only one PPLN performing ReLU activations is needed to represent all neurons, but the number of delay loops and time steps to compute each neural network layer equals the number of synapses for each neuron. This architecture may be advantageous in that it relaxes the experimental constraints for fabricating and controlling a large number of PPLNs like in the spatially-multiplexed method.

Given the relatively long ($\sim$mm) length of the PPLN, but ultrafast response time, it is desirable to employ a time-multiplexed approach for scalability. Furthermore, although we show the use of free-space coupling here, this can be eliminated through the monolithic integration of thin-film lithium niobate lasers [10], and integrated detectors [11]. This is substantially more complicated than any previously demonstrated thin-film lithium niobate photonic circuit [4, 12–14], but rapid improvements in fabrication quality/tolerance in thin-film lithium niobate photonics promise a path toward a monolithically integrated photonic neural network in the near future.

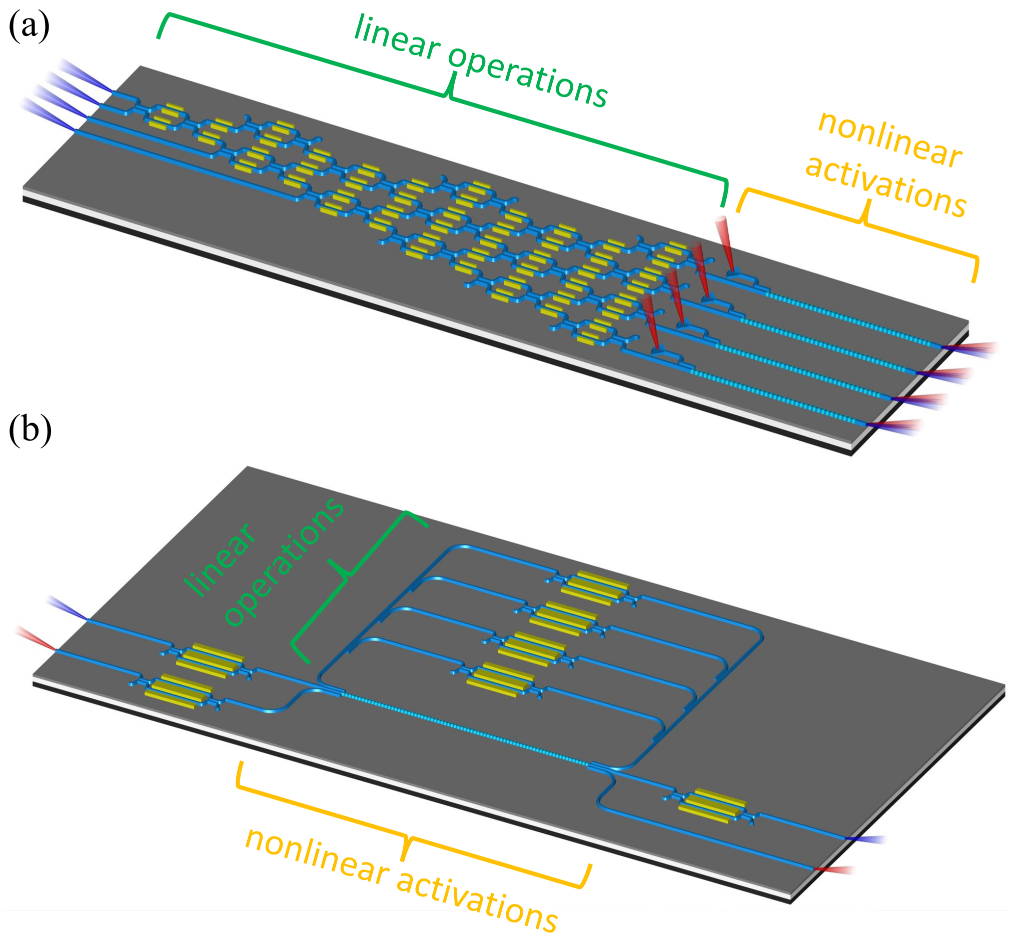

Fig. S6. Potential integrated photonic neural networks using the all-optical ultrafast ReLU function. (a) Spatially-multiplexed design based on a mesh of Mach-Zehnder interferometers performing linear operations, directly cascaded into an array of PPLNs performing the ReLU activations. (b) Time-multiplexed design based on feedback-modulated delay loops performing linear operations and the PPLN performing ReLU activations, acting as the single photonic neuron folded in time.

REFERENCES

1. M. Jankowski, A. Marandi, C. R. Phillips, R. Hamerly, K. A. Ingold, R. L. Byer, and M. M. Fejer, "Temporal simultons in optical parametric oscillators," Phys. Rev. Lett. **120**, 053904 (2018).
2. A. Marandi, N. C. Leindecker, V. Pervak, R. L. Byer, and K. L. Vodopyanov, "Coherence properties of a broadband femtosecond mid-ir optical parametric oscillator operating at degeneracy," Opt. Express **20**, 7255–7262 (2012).
3. M. A. Luda, M. Drechsler, C. T. Schmiegelow, and J. Codnia, "Compact embedded device for lock-in measurements and experiment active control," Rev. Sci. Instruments **90**, 023106 (2019).
4. L. Ledezma, R. Sekine, Q. Guo, R. Nehra, S. Jahani, and A. Marandi, "Intense optical parametric amplification in dispersion engineered nanophotonic lithium niobate waveguides," arXiv preprint arXiv:2104.08262 (2021).
5. L. Ledezma, R. Sekine, Q. Guo, R. Nehra, S. Jahani, and A. Marandi, "100 db/cm broadband optical parametric amplification in dispersion engineered nanophotonic lithium niobate waveguides," in *CLEO: Science and Innovations,* (Optical Society of America, 2021), pp. SF1C–

7.
6. C. Hu, A. Pan, T. Li, X. Wang, Y. Liu, S. Tao, C. Zeng, and J. Xia, "High-efficient coupler for thin-film lithium niobate waveguide devices," Opt. Express **29**, 5397–5406 (2021).
7. L. Deng, "The mnist database of handwritten digit images for machine learning research," IEEE Signal Process. Mag. **29**, 141–142 (2012).
8. Y. Shen, N. C. Harris, S. Skirlo, M. Prabhu, T. Baehr-Jones, M. Hochberg, X. Sun, S. Zhao, H. Larochelle, D. Englund *et al.*, "Deep learning with coherent nanophotonic circuits," Nat. Photonics **11**, 441–446 (2017).
9. F. Stelzer, A. Röhm, R. Vicente, I. Fischer, and S. Yanchuk, "Deep neural networks using a single neuron: folded-in-time architecture using feedback-modulated delay loops," Nat. communications **12**, 1–10 (2021).
10. X. Liu, X. Yan, H. Li, Y. Chen, X. Chen *et al.*, "Tunable single-mode laser on thin film lithium niobate," Opt. Lett. **46**, 5505–5508 (2021).
11. A. A. Sayem, R. Cheng, S. Wang, and H. X. Tang, "Lithium-niobate-on-insulator waveguide-integrated superconducting nanowire single-photon detectors," Appl. Phys. Lett. **116**, 151102 (2020).
12. L. Ledezma, A. Roy, L. Costa, R. Sekine, R. Gray, Q. Guo, R. M. Briggs, and A. Marandi, "Widely-tunable optical parametric oscillator in lithium niobate nanophotonics," arXiv preprint arXiv:2203.11482 (2022).
13. R. Nehra, R. Sekine, L. Ledezma, Q. Guo, R. M. Gray, A. Roy, and A. Marandi, "Few-cycle vacuum squeezing in nanophotonics," arXiv preprint arXiv:2201.06768 (2022).
14. Q. Guo, R. Sekine, L. Ledezma, R. Nehra, D. J. Dean, A. Roy, R. M. Gray, S. Jahani, and A. Marandi, "Femtojoule, femtosecond all-optical switching in lithium niobate nanophotonics," arXiv preprint arXiv:2107.09906 (2021).
